# A method for quantifying parallel growth between neuronal dendritic branches *in vitro*

**DOI:** 10.1371/journal.pone.0335919

**Published:** 2025-10-31

**Authors:** Inbar Dahari, Orly E. Weiss, Amos Ayubi, Danny Baranes, Refael Minnes

**Affiliations:** 1 Department of Molecular Biology, Ariel University, Ariel, Israel; 2 Department of Physics, Ariel University, Ariel, Israel; 3 Department of Medical Sciences, The Dr. Miriam and Sheldon G. Adelson School of Medicine, Ariel University, Ariel, Israel; University of British Columbia, CANADA

## Abstract

The morphology of dendritic trees critically shapes how neurons integrate and compute synaptic inputs. Dendritic morphogenesis results from the growth and spatial organization of branches, driven by intrinsic genetic programs, extrinsic environmental signals, activity-dependent processes, and spatial mechanisms such as tiling, avoidance, and overlap. Given their intricate architecture, particularly when branches overlap, developing methods to analyze and automate the quantification of this complexity is essential. Two-dimensional (2D) neuronal cultures provide a simplified framework for studying dendritic growth patterns but remain challenging to analyze due to network complexity, overlapping branches, and imaging limitations. Existing analysis tools often require substantial manual input or computational resources, limiting accessibility. We focused on measuring parallel growth between neighboring branches, a behavior frequently observed both in vivo and in culture. To address this challenge, we developed **SOA.2.0**, a streamlined software platform for automated segmentation and orientation analysis of dendritic branches in 2D fluorescence images. **SOA.2.0** improves the precision of morphological measurements, particularly branch parallelism, while remaining adaptable across diverse cellular and network models. Using **SOA.2.0**, we quantified the extent of parallel growth among dendritic branches in cultured hippocampal neurons and compared these measurements with simulated random branch distributions. Our analysis revealed that parallel growth is a prevalent and non-random phenomenon, occurring among both sister and primarily non-sister branches of all generations, with frequencies significantly exceeding those observed in simulated random distributions. This behavior was frequently observed in relatively large groups of branches, sometimes up to eight, that extended for dozens of microns. Notably, this pattern was not detected in astrocytic processes within the culture. These results indicate that parallel branch growth is a prominent feature of dendritic architecture and may contribute to shaping the structural organization of neuronal networks, offering new insights into the mechanisms underlying their development and function.

## Introduction

The development and structure of dendritic arbors play a crucial role in determining how neurons process input and contribute to overall network activity [1–5]. The intricate structure of dendritic trees is critical for neural circuit formation and function, influencing the types and locations of inputs a neuron receives and how these inputs are integrated [6–8]. This complex process involves multiple stages, including branch initiation, outgrowth and guidance, branching, synapse formation, and stabilization [9,10]. It is regulated by a diverse array of intrinsic genetic programs and extrinsic environmental cues [11–15]. including growth factors, adhesion molecules, and cytoskeletal dynamics, as well as a combination of activity-independent signals and activity-dependent synaptic plasticity [16,17], which together guide and balance the spatial and temporal patterning of dendritic arborization [18]. Deviations in this balance have been implicated in both degenerative and neuro-developmental disorders [19,20].

Another key mechanism of dendritic arborization involves spatial organization and overlapping relationship between neighboring dendritic branches. Dendritic arbors from functionally similar neurons innervating a shared territory can form minimally overlapping dendritic or axonal fields, known as tiling [21]. This spacing mechanism ensures that arbors maximize their coverage of a given territory while minimizing redundant innervation. Parallel growth among dendritic branches is a common manifestation of this spacing strategy. For example, in the hippocampus, individual dendritic trees exhibit parallel functional architectures, enabling distinct processing of synaptic inputs [22]. This parallelism is further reflected in the organization of hippocampal circuits, which are segregated into specialized pathways that facilitate differential information processing, thereby enhancing learning and memory formation [23].

In contrast to the parallel growth, adhesive interactions between dendritic arbors can help maintain the coherence of dendrites at specific target sites [24], or facilitate the bundling of functionally similar processes, potentially coordinating their activity [25]. Altogether, these insights highlight the importance of understanding and quantifying how dendritic branches either avoid or interact with each other, as this can provide key insights into the functional logic underlying neural circuit organization.

However, due to the complexity of neuronal networks, extensive software-based analysis is required for automated and high-content data extraction from neuronal network images. Numerous tools (e.g., Neurolucida, TREES Toolbox, Simple Neurite Tracer, Imaris Filament Tracer) have been developed for the automatic tracking and analysis of dendritic branches in fluorescently labeled nervous tissue and cells [26–29]. These tools offer a range of functions, including segmentation using open-source software, quantitative axonal analysis via Blender-based tools, and fractal analysis methods. While these advanced tools provide valuable insights into dendritic morphology, they are often optimized for 3D single-cell reconstructions or require user-guided tracing and commercial software.

In addition to the complexity of existing tools for dendritic structure analysis, segmenting 3D images of dendritic arbors using advanced software remains challenging due to the intricate and compact nature of dendritic trees, variations in their morphology, and limitations such as noise, artifacts, and restricted imaging resolution. Furthermore, overlapping dendrites, intensity variations, and the need for manual intervention further complicate segmentation, requiring advanced algorithms and substantial computational resources. Given these challenges, examining the structural features of dendritic trees in two-dimensional (2D) neuronal cultures presents a practical and simplified alternative. However, even in 2D cultures analysis remains challenging due to the complexity of dendritic network formation.

Typically, analyses of dendritic tree structure focus on individual branches without considering their relationship to surrounding branches [5,30]. In contrast, our research examines dendritic trees, their branches, and their development in the context of neighboring branches. An example of this approach is demonstrated in the work of Cove et al [31], which shows that dendritic branches tend to grow toward intersections formed by the branches of neighboring dendritic trees.

We found that dendritic branches in hippocampal neuronal cultures frequently grow in parallel trajectories without intersecting, suggesting a spatial arrangement that minimizes overlap. This phenomenon occurs between both sister and non-sister dendritic branches and is independent of synaptic activity. To determine whether this dendritic branch’s behavior in culture is directed or random and to quantify its frequency and extent, we utilized in-house software alongside random simulation models.

We developed SOA (Segmentation and Orientation Analysis), a simple automated tool for direct segmentation and orientation analysis of dendritic branches within neuronal networks [32]. This study utilized SOA.2.0, an updated software version, to segment dendritic branches in 2D fluorescence images of cultured dendritic networks and to characterize their morphological properties. The software accurately detects the dendritic branches and performs measurements of morphological parameters such as growth in parallel trajectories. Key functions include real-time threshold adjustment and segmentation of dense 2D cultures, automated classification of parallel branch groups, and a lightweight graphical user interface (GUI) designed for accessibility to users without programming expertise or high-performance computing resources. SOA.2.0 also exhibits a high level of adaptability for examining cellular mechanisms in various cell categories as well as for investigating non-biological network systems.

By quantifying the extent of parallelism among dendritic branches in culture using SOA.2.0 and comparing it to simulations of randomly distributed branches, we conclude that branch-to-branch parallel growth is a prevalent and non-random phenomenon. We present a model illustrating how dendritic arborization architectures are shaped by parallel growth among non-sister branches. The findings of this study establish a quantitative framework for understanding parallel-growth-driven dendritic morphogenesis, providing insights into the mechanisms that govern neuronal network development and function.

## Materials and methods

### Image analysis using the SOA.2.0 software

#### Detection and segmentation of dendritic branches.

**Fig 1:**
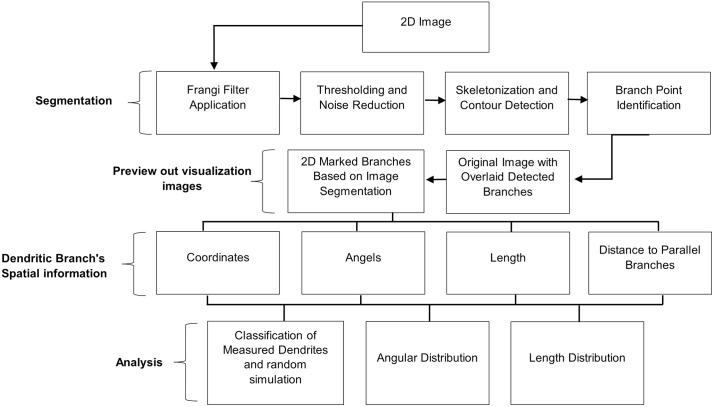
The SOA.2.0 workflow for segmentation and growth direction analysis is depicted. The 2D image is uploaded and segmented, allowing the extraction of spatial information for each dendritic branch. The collected data supports the analysis of morphological parameters.

Segmentation was applied to identify dendritic branches and separate them from the background (Fig 1). A user-friendly graphical user interface (GUI) was developed using Tkinter to facilitate image loading and interaction through file dialogs.

Images were preprocessed by converting them to grayscales and applying the Frangi filter to highlight ridge-like structures characteristic of dendritic branches. The Frangi filter was implemented using Python libraries like NumPy for matrix operations. A range of sigma values (0.25 to 3.5, incremented by 0.25) was used to capture varying branch widths. Dynamic thresholding allowed users to adjust the threshold through a slider for real-time optimization. Small, irrelevant objects were removed to retain significant dendritic branches only. Skeletonized images were analyzed for morphological features such as length and connectivity.

#### Contour detection and feature extraction.

**Fig 2:**
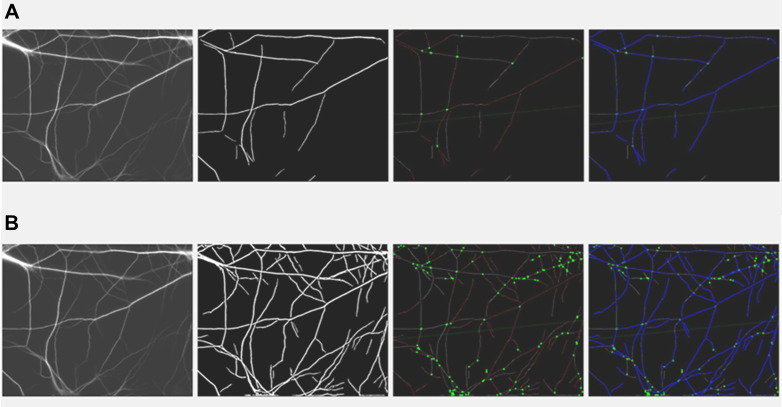
GUI-based adjustments for threshold and branch length. (A) Default threshold and branch length values; (B) Adjusted threshold and branch length values for optimal segmentation.

Contours representing the boundaries of dendritic branches were detected, with branch points identified by pixel connectivity analysis. Branches shorter than a user-defined threshold were excluded to focus on significant structures. The branch length threshold could be adjusted via the GUI for targeted analysis (Fig 2).

The enhanced segmentation process not only allowed for the detailed examination of dendritic structures but also supported the comprehensive analysis of their morphological parameters. This approach facilitated the accurate quantification of dendritic orientations and branch connectivity, providing essential data for analyzing neuronal network structure. All processed images, including original, segmented, and skeletonized outputs, were saved directly from the GUI for further analysis and documentation.

#### Data collection and analysis.

Data regarding the location, angle, and length of each detected branch was collected. Analyses included dendritic branch classification (parallel detected by SOA.2.0 vs. random simulation), length distribution, and angular distribution. Length measurements were in pixels, and angles were measured in degrees relative to the horizontal axis. The shortest distance between dendritic branches was also determined.

#### Random simulation.

To determine if the observed degree of parallelism is greater than what would be expected by random chance, we simulated a null model of randomly oriented branches. In each simulation, the number of segments (n) matched the number of branches in the original image. Each segment was assigned a random angle drawn uniformly from the range of 0°–180°, without directional bias or interaction. Spatial positions were not simulated, as only angular relationships were assessed.

The probability of “success” (i.e., the chance that two branches fall within a narrow angular window and are therefore considered parallel) was estimated as p=1/n. This choice reflects the assumption that as the number of branches increases, the probability of any two segments randomly aligning within a narrow angular threshold (±10°) decreases proportionally. This inverse relationship accounts for image complexity and prevents overestimation of parallelism in denser images.

We modeled the likelihood of observing k parallel branches within each group using the binomial distribution formula as described in Dahari et al.:


$$P=\;\frac{n!}{k!\times (n-k)!}\times p^{k}\times {\left(1-p\right)}^{n-k}$$

$$P=\;\frac{n!}{k!\times (n-k)!}\times {\left(\frac{\text{1}}{n}\right)}^{k}\times {\left(1-\frac{\text{1}}{n}\right)}^{n-k}\times n=\frac{n!}{k!\times (n-k)!}\times {\left(\frac{\text{1}}{n}\right)}^{k-1}\times {\left(1-\frac{\text{1}}{n}\right)}^{n-k}$$ (1)

To simplify interpretation and allow direct comparison with empirical results, we multiplied the binomial probability P(k) by the total number of branches (n), yielding the expected count of parallel branches for each group size. This provides a baseline expectation for parallelism in a randomly oriented system with identical complexity to the biological image.

#### Software components.

SOA.2.0 was developed using the following Python libraries: *OpenCV* for computer vision tasks, *NumPy* for scientific computing, *Matplotlib* for 2D plotting, *scikit-image* for image processing, *PIL* for image display, *Pandas* for data manipulation and CSV export and *Tkinter* for GUI creation. The interface allowed users to upload images, adjust segmentation settings, and view segmented results in real-time.

The full source code, user guide, and example dataset for SOA.2.0 are publicly available on GitHub at: https://github.com/inbar2748/SOA-2.0. This repository allows users to run the software, test it on sample data, and reproduce the results described in this study.

#### Hippocampal cell culture.

Cultures of dissociated hippocampal cells were prepared according to the methods of Weiss and colleagues [33]. Briefly, hippocampi of mouse pups (0–4 days old) were dissected and dissociated using trypsin (0.25%, Sigma, USA). The cells were maintained in a recovery medium consisting of 85% Minimal Essential Eagle’s Medium (MEM, Sigma, USA), 10% inactivated fetal bovine serum (iFBS, Sigma, USA), 2% D-glucose (Thermo Fisher Scientific, USA), and 1% L-glutamine (Thermo Fisher Scientific, USA). Dissociated cells ($4\times 10^{\text{4}}$ cells/ml) were seeded to coral slices. After 24 h, the medium was replaced with a supplemented culture medium consisting of 45% MEM, 40% Dulbecco’s Modified Eagle Medium (DMEM, Sigma, USA), 10% nutrient mixture F-12 Ham formulation (Sigma, USA), 0.34% D-glucose, 2% B-27 supplement (Thermo Fisher Scientific, USA), 0.25% (w/v) bovine serum albumin (Sigma, USA), 0.25% L-glutamine, and 0.01% kynurenic acid (Thermo Fisher Scientific, USA). The cells were further incubated (37°C, 10% CO_2_) for 1–3 weeks.

#### Immunofluorescence staining..

Cultures were fixed with paraformaldehyde (4%; Sigma, USA) followed by incubation for 10 min with 0.25% Triton X-100 (TEDIA, USA), then further incubated for 1 h in 3% Inactivated Goat Serum (INGS). Primary antibody staining was performed using 0.0025 mg/mL anti-MAP2 (Microtubule associated protein 2) antibody, or an antibody against the astrocytic marker protein Glial Fibrillary Acidic protein (GFAP), overnight at 4°C. Both MAP2 and GFAP are well-established markers of dendrites and astrocytes (respectively), showing minimal to no staining of axons. Similarly, anti-GFAP is a strong and specific label of astrocytes. Secondary antibody staining was performed by 1 h incubation (room temperature) with 0.002 mg/ml Polyclonal Goat ant mouse (green, Alexa 488, for labeling anti-MAP2), and goat anti rabbit (Red, Alexa555, to label anti-GFAP). Samples were mounted in elvanol and 1,4-diazabicyclo[2.2.2]octane)DABCO(2.5%.

#### Microscopy.

Fluorescent images were acquired using an inverted Zeiss Axio-observer Z1 microscope equipped with X20/0.45 and X40/0.10N.A., and with a fluorescent FITC and Rhodamine filter cubes.

#### SOA.2.0 graphical user interface (GUI).

**Fig 3:**
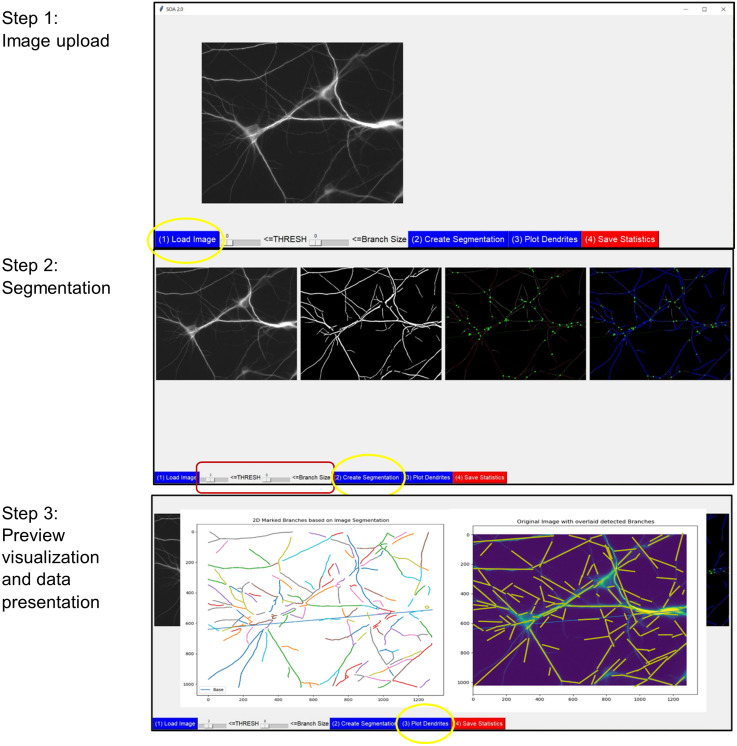
GUI workflow. Step 1: Image upload; Step 2: Parameter adjustment; Step 3: Branch detection and analysis.

The GUI steps for processing an image are outlined in Fig 3 and are designed to enable intuitive interaction with each stage of the analysis pipeline. In Step 1, users can upload 2D fluorescence microscopy images in commonly supported formats including JPG, PNG, TIFF, and BMP. Only single-file uploads are supported per instance, and upon selection, the image is previewed in real time within the interface. In Step 2, users can adjust two key segmentation parameters through interactive sliders. The first slider controls the grayscale intensity threshold used for binary segmentation, where values from 1 to 10 correspond to predefined intensity thresholds mapped to the 0–255 grayscale range. Lower slider values increase sensitivity to faint dendritic structures, while higher values emphasize stronger, more prominent features. The second slider determines the minimum branch length required for a segment to be included in the analysis. Slider values from 1 to 10 correspond to length thresholds ranging from 5 to 200 pixels. If no value is selected, a default threshold of 50 pixels is applied. In Step 3, the results of the segmentation and analysis are visualized in real time within the GUI. One display overlays the skeletonized dendritic branches on the original image, with accepted branches, filtered by the user-defined length threshold, highlighted and annotated. A second display presents the spatial distribution of detected segments, with each branch color-coded by its group assignment (e.g., based on orientation). Users can then export the analysis results, including raw measurements, summary statistics, and annotated images, in both CSV and PNG formats.

#### Displaying output results.

**Fig 4:**
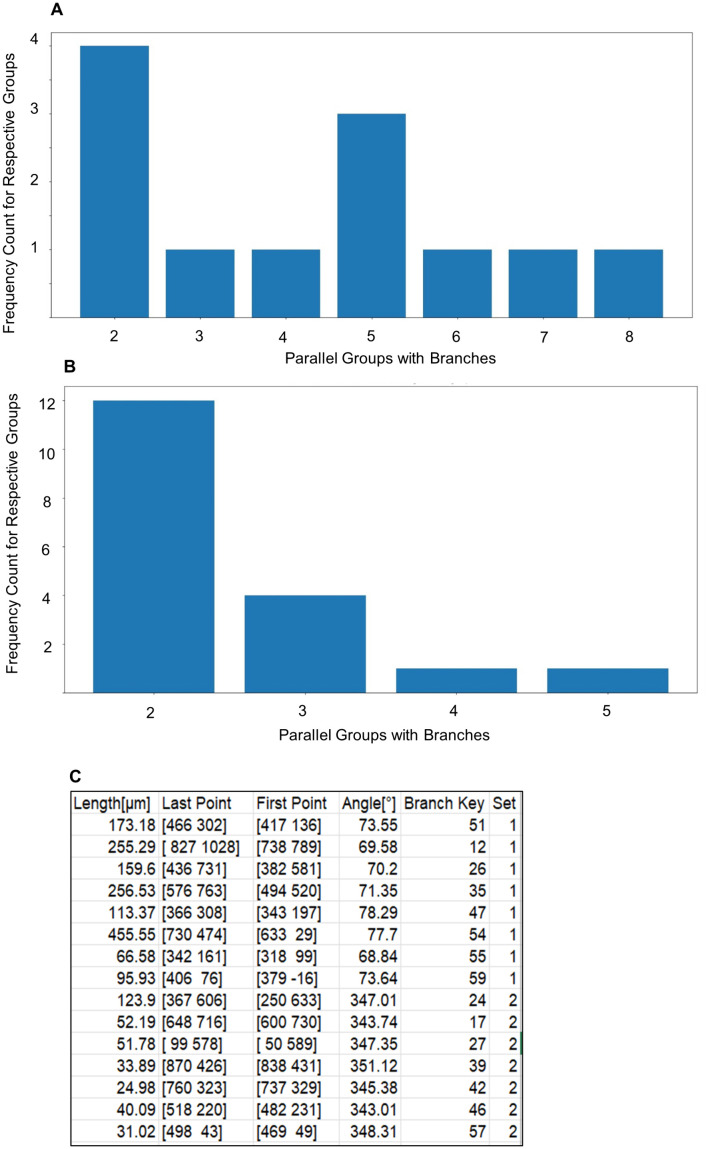
Classification of dendritic branch parallel growth vs. random simulation. (A) Dendritic branches that were detected by SOA.2.0 were classified by angle range as parallels and divided into groups of pairs/triples/quartets and so on. The frequency count for respective groups is labeled as “Parallel Groups with Branches,” with the number of detected total branches equal to 61 and arranged into 12 groups. (B) Dendritic branches that were calculated by simulation of random growth and were classified by angle range as parallels and divided into groups of pairs/triples/quartets. It shows 61 branches arranged into 18 groups. The occurrence frequency of each group is shown in the graph. (C) A table containing information about parallel groups.

A total of 98 fluorescent images were processed with SOA.2.0, and results were classified into parallel groups (Fig 4). Non-parallel segments were analyzed for angle flexibility, limited to a maximum deviation of 10°. Results were compared to simulations of random line distributions.

### Statistical analysis

SOA.2.0 facilitated data fusion from multiple images for comprehensive comparison and statistical analysis. Individual image data was aggregated, and statistical analyses were conducted to measure parallel growth.

### Calculation of parallelism percentage

The parallelism percentage is a key metric used to quantify the growth of dendritic branches. This analysis involved calculating the parallelism percentage for both the empirical data and the simulated data generated by the computational model. These percentages allow us to compare the structure and behavior of dendritic branches between real and simulated data.

### Measured parallelism percentage calculation

For the measured data, the total number of parallel groups is identified from the experimental images of dendritic branches. Each parallel group is defined as a set of branches that grow in close alignment with one another. The frequency of each parallel group size (i.e., the number of branches that form a group) is counted, and the percentage of Measured Parallelism (PM) is calculated using the following formula:


$$PM=\frac{\sum \left(Number\;of\;Groups\times Frequency\;of\;Each\;Group\right)}{Total\;Number\;of\;Detected\;Branches\;in\;the\;Image}$$ (2)

Where:

- The number of groups refers to the size of each parallel group (i.e., how many branches are aligned together).
- The frequency represents how often each group size occurs.
- The total number of detected branches is the total number of dendritic branches identified in the image.

The result is a percentage that reflects the extent of parallel alignment observed in the biological measurements.

### Simulation parallelism percentage calculation

Similarly, for the simulated data, we calculated the percentage of Simulation Parallelism (SP) using the predicted data generated by the computational model. The computational model simulates dendritic growth and predicts how many branches form parallel groups.

The simulation parallelism percentage is computed using the same approach as for the measured data, with the following formula:


$$PS=\frac{\sum \left(Simulated\;Number\;of\;Groups\times Frequency\;of\;Each\;Simulated\;Group\right)}{Total\;Number\;of\;Detected\;Branches\;in\;the\;Image}$$ (3)

Where:

- The simulated number of groups represents the predicted size of each parallel group from the simulation.
- The frequency represents how often each group size occurs in the simulated data.
- The total number of detected branches is the same as in the measured data, to ensure consistency in comparison.

### Ratio between measured and simulated data

To assess the accuracy and performance of the computational model, we calculated the ratio between the Measured and Simulated Parallelism (PMS) percentages. This ratio provides insight into how well the model reflects the actual biological data. The ratio is calculated using the formula:


$$PMS=\frac{\sum \left(Measured\;Number\;of\;Groups\times Frequency\;of\;measured\;Group\right)}{\sum \left(Simulated\;Number\;of\;Groups\times Frequency\;of\;Simulated\;Group\right)}$$ (4)

This ratio (denoted as PMS) quantifies the degree to which the measured data aligns with the simulation. A value close to 1 indicates that the model closely mirrors the measured data, while values greater or less than 1 suggest discrepancies between the two.

### Calculation of group-weighted parallelism

We introduced “Group-Weighted Parallelism” to assign a higher value to parallel lines that are part of larger groups. The Group-Weighted Parallelism is calculated by assigning weights based on the size of the group in which a parallel line is found. Specifically, the weight of each group is multiplied by the group size. The formula for this calculation is:


$$Weighted\;parallelism\;sum=\sum ({Group\;Size}^{\text{2}}\;\times \;Frequency\;of\;Each\;Group)\;\;\mathrm{\backslash ]}$$ (5)

- Group Size represents the number of parallel lines in a group.
- Frequency of Each Group refers to how often each group size occurs in the data.

We apply this calculation to both the measured data (from biological observations) and the simulated data (from the computational model). By comparing the weighted sums of these two datasets, we derive the Group-Weighted Parallelism ratio (Measured/Simulation), which represents how well the simulation aligns with the measured data, particularly with respect to larger parallel groups. The formula for the Group-Weighted Parallelism ratio (M/S) is:


$$M/S=\frac{\sum Weighted\;parallelism\;(Measured)\;}{\sum Weighted\;parallelism\;(Simulated\;)\;}\;\mathrm{\backslash ]}$$ (6)

- A ratio greater than 1 indicates that the measured data contains more Group-Weighted Parallelism (particularly in large groups) than the simulation predicts.
- A ratio less than 1 suggests that the simulation overestimates Group-Weighted Parallelism relative to the measured data.

### Software validation

Accuracy was verified by comparing SOA.2.0 results with manually annotated images. Reliability was tested across multiple images of the same neuronal cultures. Quality assurance included extensive testing cycles for bug detection and tool stability to ensure readiness for research use.

## Results

### Parallel growth of sister- and non-sister dendritic branches in culture

**Fig 5:**
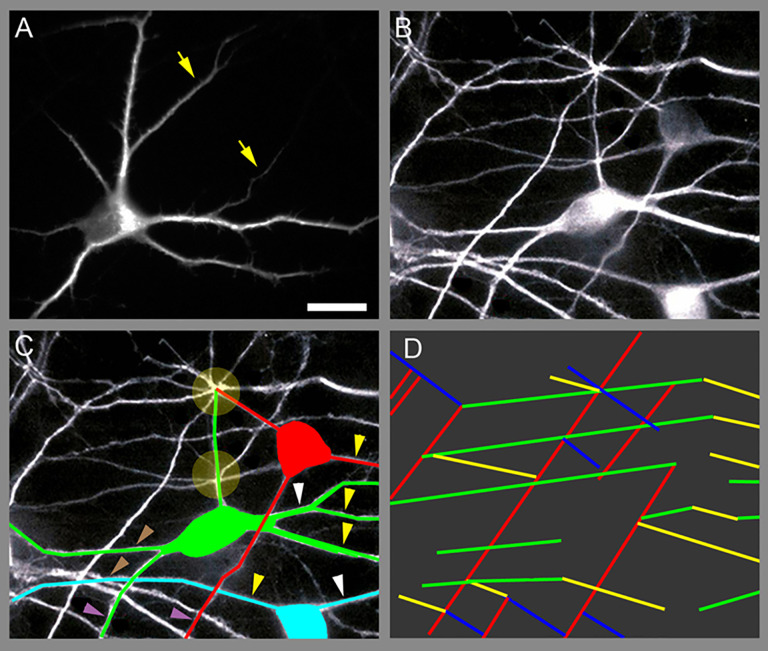
Parallel growth among sister- and non-sister dendritic branches. Representative images taken from 7 days old hippocampal neuronal cultures immunolabeled with an antibody against MAP2. (A) An isolated neuron. Arrows indicate parallel sister dendritic branches. (B) A network of neurons. (C) Full neurons are color-coded. White dendritic branches belong to neurons with cell bodies located outside the field of view. Circles mark sites of branch-to-branch adhesion. Arrowheads, color-matched to corresponding branches, indicate parallel dendritic (D) Schematic representation of the parallel branches in (C), with each parallel group assigned a unique color. Scale: 15 mm.

Hippocampal neuronal cultures labeled with an antibody against the dendritic marker MAP2 revealed a distinct pattern of parallel growth among dendritic branches of all generations. This behavior was observed between sister dendrites, which emerged mostly from single neurons grown in relative isolation (Fig 5A). In denser regions of the culture, parallel growth predominantly occurred among non-sister dendritic branches (Figs 5B and 5C). Parallel growth extended over distances of up to several dozen microns (Fig 5C) and was frequently (Fig 5D). A high proportion of dendritic branches exhibited parallel growth, forming groups containing between 2 and 8 branches (Fig 5D, see also Fig 4).

### Data processing, collection, and analysis

Upon obtaining a significant dataset of images, we uploaded it into the software to evaluate various parameters for each image, including the number of dendrite branches, number of parallel dendrites and number of parallel groups (S1 Table). We utilized statistical methods to analyze the images, extracting insights and deriving conclusions from the data.

In the initial phase, we performed additional calculations and processing on the data obtained from the software for each image separately. This involved adding a calculation of “Group-Weighted Parallelism” to both the random case (simulation) and the case measured by SOA. To achieve this, we developed a Python program tailored to process the information for each image.

Subsequently, we gathered and consolidated the data extracted from the SOA software from 98 existing images into a single database (S2 Table). The program systematically traverses folders and files, collecting the necessary data for performing statistical analysis. We ensured that the data was organized and that any summarized information was linked back to its source, facilitating further investigation if needed.

In the third step, we presented the selected data visually on a graph to substantiate the research claim. Through this visual representation, we observed a noticeable difference between the random simulation data and the data measured by the software.

### Non-sister dendritic branches grow in parallel in a non-random manner: Comparison of measured and simulated parallel growth results

Each fluorescence image was analyzed separately utilizing the SOA.2.0 software to detect dendritic branches and quantify morphological parameters, such as the percentage of parallelism and spatial distribution of branches. These metrics provided a comprehensive characterization of dendritic network structures at the single-image level, enabling the identification of localized growth patterns specific to each sample.

### Total number of branches

Across 98 samples, the total number of detected dendritic branches was analyzed in both measured and simulated data. The mean number of detected branches was 109 with a standard deviation of 44, indicating variability across the samples. This count forms the baseline for calculating parallelism percentages in both measured and simulated scenarios.

### Parallel groups

The measured data identified 17 parallel groups (standard deviation: 5). The simulation predicted 31 parallel groups (standard deviation: 12), which is nearly double the measured count. This discrepancy indicates that the binomial distribution model overestimates the number of groups, potentially suggesting a more fragmented or less aligned structure than is observed.

### Comparison of measured and simulated parallelism percentage

**Fig 6:**
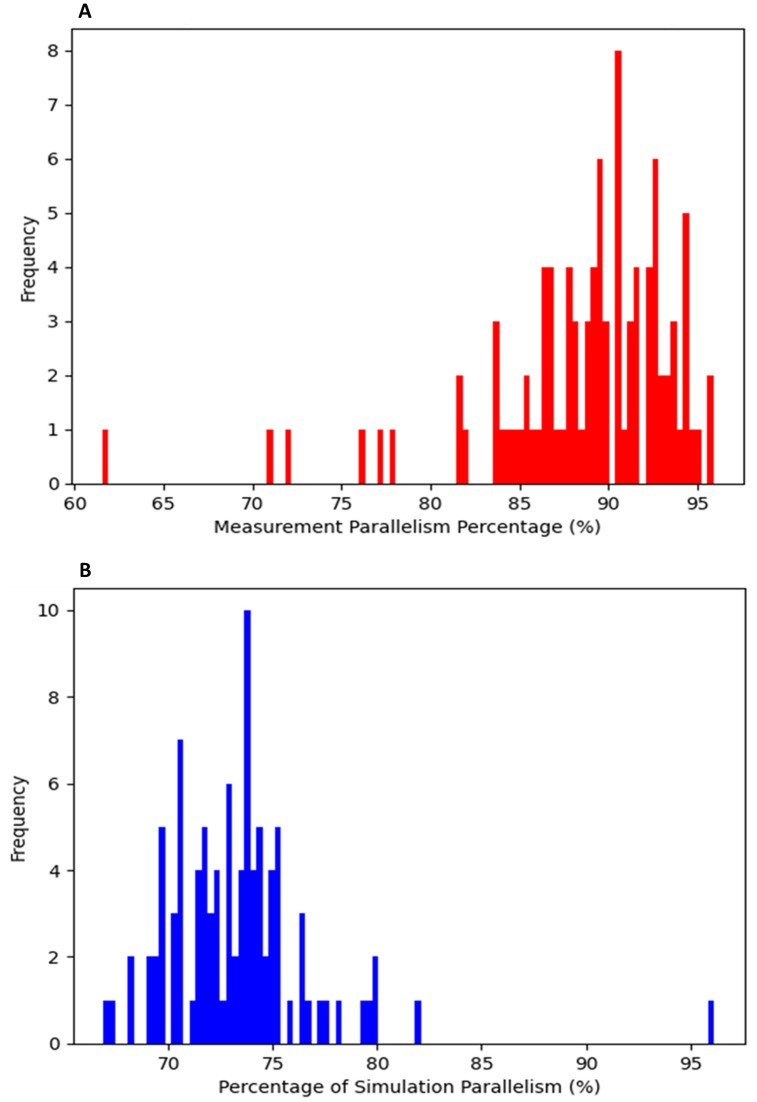
Distribution of parallelism percentages. This figure presents a comparison between measured and simulated parallelism for all 98 analyzed images. The x-axis represents the parallelism percentage (%), while the y-axis represents the frequency of occurrence for each percentage. (A) Histogram of parallelism percentages obtained from measurements. (B) Histogram of parallelism percentages derived from simulations.

Fig 6 illustrates the distribution of parallelism percentages, quantifying the degree of alignment and structural organization of dendritic branches in both measured and simulated data. The histogram in Fig 6A shows that most measured parallelism values range between 85% and 95%, with the highest frequency occurring around 90% (peak frequency of 8). A few outliers exhibit lower parallelism percentages, reaching as low as 65% (S2 Table), but these cases are rare. In contrast, Fig 6B presents the simulation results, where the majority of parallelism percentages are concentrated between 70% and 75%, with a peak frequency of 10 at approximately 75%. This indicates that the model predicts a lower degree of alignment compared to actual measurements. While the simulation rarely produces parallelism values above 85%, the measured data frequently reaches higher percentages. The average measured parallelism percentage (PM) was 89% (standard deviation: 5%), whereas the simulated parallelism percentage (PS) was 73% (standard deviation: 4%). The notable discrepancy between the parallelism percentage values of the measured and simulated suggests that the simulation underestimates the degree of parallelism, potentially due to oversimplified assumptions or inaccuracies in modeling the biological growth process.

### Measured-to-simulation ratio (PMS)

The measured-to-simulation ratio (PMS) provides a quantitative comparison of parallelism between the two datasets. The mean PMS value was 1.213, indicating that the measured data exhibited 21.3% more parallelism than the simulated data

### Parallelism distribution and regression analysis

**Fig 7:**
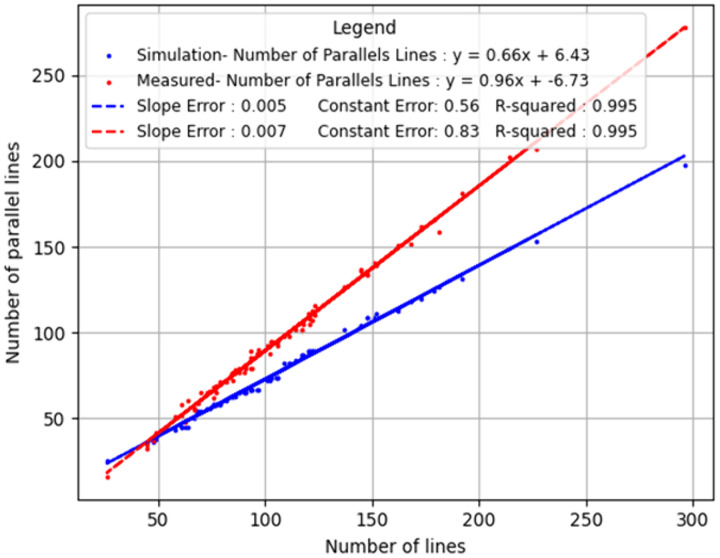
Comparison of parallel line detection in measured and simulated data. The x-axis represents the total number of lines detected by the software, while the y-axis indicates the subset of those lines classified as parallel. The blue dots illustrate the expected number of parallel lines based on a binomial random distribution, whereas the red dots depict the actual number of parallel lines identified in the measured dataset.

Fig 7 presents a comprehensive comparison between the number of detected lines and the number of lines classified as parallel. The red dots, representing data from 81 images, correspond to the detected structures using the SOA software. A linear relationship is observed in both cases because, mathematically, the number of potential branch-pair comparisons increases proportionally with the total number of branches. In the simulation, this yields a baseline linear trend with a slope of approximately 0.66. In the measured data, however, the slope is significantly steeper (≈0.96), indicating that a higher proportion of branches form parallel relationships than would be expected by chance alone. This contrast in slopes supports the conclusion that the biological growth pattern involves non-random, spatially coordinated alignment among branches.

The high linearity (R ≈ 0.995 for both datasets) suggests that parallelism scales reliably with branch count, but the biological samples consistently demonstrate enhanced parallel growth compared to the random model. This difference is visually and quantitatively evident from the divergence in slope between the red (measured) and blue (simulated) lines.

### Statistical significance and regression analysis

To assess the statistical significance of the results, a regression line was fitted to each distribution, enabling a standardized metric across all data points. The R coefficient—indicating the degree of linear fit—was determined to be R = 0.9952 for the measured data and R = 0.9955 for the random distribution, demonstrating a strong correlation. The slope of the regression line (referred to as the “weight”) represents the degree of parallelism. In the random distribution, the slope was w = 0.66, while in the measured data analyzed using the SOA software, the slope was w = 0.96. The calculated ratio between these values is 1.45, indicating that the proportion of parallel lines in dendritic branch images is approximately 45% higher than in the random distribution.

### Group-weighted parallelism analysis

**Fig 8:**
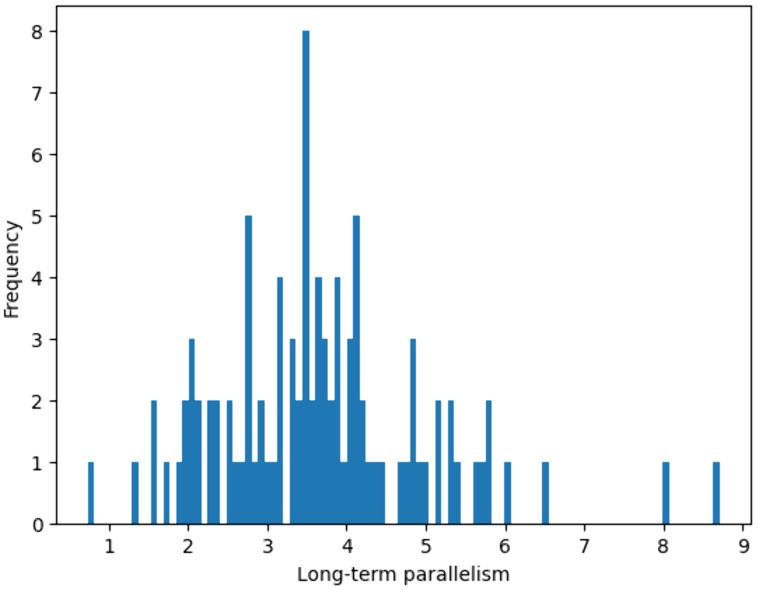
Distribution of the group-weighted parallelism ratio (E/S). The x-axis represents the Group-Weighted Parallelism ratio, comparing measured data (E) relative to simulated data (S). The y-axis represents the frequency of each ratio across the dataset.

The distribution of Group-Weighted Parallelism ratios predominantly clusters around 3–4, indicating that, in most cases, the measured data exhibits approximately three to four times the contribution of larger parallel groups compared to the simulation (Fig 8). However, a few instances of lower ratios (close to 1) suggest that in some cases, the simulation closely approximates the measured data in terms of Group-Weighted Parallelism.

**Fig 9:**
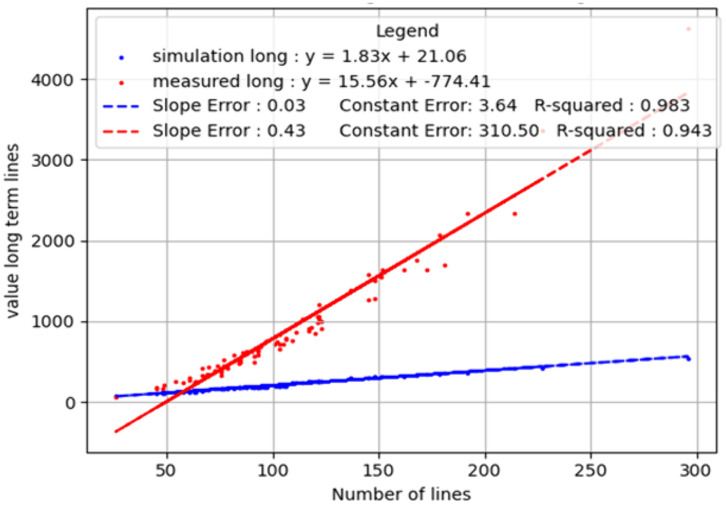
Summary of the data comparisons. The red dots represent the data detected by the SOA software, while the blue dots represent the expected number of parallel lines according to the binomial normal distribution.

Fig 9 provides a comprehensive comparison of Group-Weighted Parallelism values detected by SOA software versus the corresponding values derived from a binomial random distribution.

A regression analysis was performed to assess the relationship between measured and simulated data. The R coefficient, which quantifies the degree of linear fit, was found to be R = 0.983 and R = 0.943, indicating a strong correlation between the dataset and the model. The slope of the regression line, referred to as the “weight”, represents the level of parallelism. In the random case, the weight was w = 1.83, while in the SOA-measured data, it was significantly higher at w = 15.56.

To further assess the applicability of SOA 2.0 to different cell types, we analyzed astrocytic processes in co-culture with neurons (data shown in the supplementary). In contrast to dendrites, which showed clear and statistically significant parallel alignment, the astrocytic processes did not exhibit comparably strong or significant parallel organization. A Mann–Whitney U test indicated no statistical difference between the measured astrocyte parallelism and the simulated random baseline (p = 0.007, see S3 Table).

In addition to the astrocyte control analysis, we further evaluated the performance of SOA 2.0 using digitally reconstructed neurons and glia obtained from the public NeuroMorpho.Org repository. This dataset included two representative images of neuronal morphologies reconstructed under experimental conditions distinct from those used in our cultured networks. The analysis revealed that measured parallelism was slightly lower than the simulated random baseline, with an average measured-to-simulated ratio of 0.88 and an E/S weight ratio of 0.95 (see S4 Table). These findings are consistent with a predominantly random orientation of dendritic branches in isolated neuron reconstructions. When considered alongside the astrocyte control data, this external validation further supports the specificity of the parallel alignment observed in dendritic cultures and demonstrates that SOA 2.0 reliably detects biologically meaningful parallelism without artificially generating alignment in datasets lacking structured organization.

## Discussion

The innovation and significance of our work lies in the development of SOA 2.0, an advanced tool that automates the segmentation and analysis of dendritic branches while introducing a novel method for quantifying morphological properties such as parallelism and spatial distribution. Building on a limited, previously published prototype (SOA 1.0) that lacked flexibility, SOA 2.0 was designed as a substantial improvement, developed initially to address our specific experimental needs but intentionally structured for broad applicability. By leveraging this enhanced approach, we systematically evaluate our hypothesis regarding the structured, non-random nature of parallel dendritic expansion, offering fresh insights into the architectural organization of neuronal networks.

### Characteristics of parallel growth in dendritic branches indicate it is not a random phenomenon

Our findings suggest that sister dendritic branches in neuronal cultures often grow in parallel, indicating an intrinsic regulatory mechanism in dendritic development. Since sister branches originate from the same parent dendrite, they may share similar molecular cues and growth factors that guide their parallel growth [34]. However, the observation that non-sister dendritic branches exhibit even higher levels of parallel growth is particularly intriguing. This suggests that extrinsic factors, such as local environmental cues, interactions with neighboring neurons, and extracellular signaling molecules, significantly contribute to parallel growth [35]. The ability of non-sister branches to align in parallel implies a degree of coordination and communication across multiple neurons, potentially mediated by adhesion proteins and other molecular guidance mechanisms [36].

### Extent and spatial constraints of parallel growth

Our analysis reveals that parallel dendritic growth can extend up to 50 micrometers, underscoring the potential for extensive coordination in dendritic patterning. However, this distance may represent an upper limit, constrained by factors [37], diffusion limits of signaling molecules that regulate growth and mechanical properties of the dendrites themselves. It also suggests that proximity among the branches is a key factor in this process. Branches that are closer together are more likely to be influenced by the same localized signaling cues and be engaged in direct physical interactions or near-contact communication.

The extent to which dendrites were involved in parallel growth also suggest that parallel growth is non-random. The finding that more than 20% of the branches participate in parallel growth indicates that this phenomenon is common, though not universal. This partial involvement may indicate that parallel growth is driven by selective molecular cues, making it a regulated, rather than stochastic, process. It suggests that dendrites employ multiple strategies for spatial organization, with parallel alignment being just one among several. Moreover, the fact that single set of branches can contain up to eight parallel branches from multiple dendritic trees, highlights the potential for complex, coordinated growth patterns that contribute to the structural organization of neuronal networks.

In addition to the above, the extent of parallel growth in cultured dendritic branches is significantly greater than what is predicted by a random line distribution model. The measured data exhibited 21.3% greater parallelism than the simulation (PMS ratio), suggesting that the model underestimates the degree of parallel growth. This underestimation likely arises from oversimplified assumptions about dendritic growth dynamics and from failure to account for complex biological interactions. Altogether, these findings emphasize that parallel dendritic growth is an actively regulated process, shaped by molecular and cellular factors that extend beyond simple geometric constraints.

### The dendritic parallel growth is neuron-specific

To ensure that the observed results reflect biologically meaningful organization rather than artifacts of image processing or random alignment, we performed a control analysis using images of astrocytes co-cultured with neurons. As shown in the Supporting information (S3 Table), this analysis revealed minimal parallelism, reinforcing the specificity of the findings observed in dendritic cultures and supporting the interpretation that parallel alignment is a distinctive feature of neuronal dendritic organization.

### Variability in measured vs. simulated data

The variability in measured and simulated data provides further insights into dendritic growth dynamic. Measured parallelism exhibited higher variability (5%) compared to the simulation (4%), suggesting that real dendritic growth is inherently more variable than the controlled predictions of the model. Also, the number of detected lines also showed greater variability in measured data (standard deviation = 43) compared to the simulation (standard deviation = 29), reinforcing the idea that the simulation is more deterministic and may oversimplify the branching behavior. These differences indicate that biological dendritic growth is influenced by additional stochastic or regulatory mechanisms, which are not fully captured by the current simulation model.

### Importance of quantifying parallel growth

The development of SOA 2.0 as a quantitative tool for assessing dendritic parallelism represents a significant advancement in understanding neuronal network architecture. By enabling precise measurement and analysis, this tool allows researchers to identify the factors influencing dendritic development, and to understand how the brain processes and consolidates information through organized dendritic patterning. It also enables to investigate potential disruptions in neurodevelopmental disorders, where dendritic architecture may be altered. Hence, quantifying parallel growth is essential for elucidating the mechanisms governing synaptic connectivity and network functionality, providing a foundation for further research into neuronal circuit formation.

### Impact on dendritic arborization

Parallel growth among non-sister branches plays a critical role in shaping dendritic arbors. By growing in parallel, these branches create organized, non-overlapping patterns, optimizing space for synaptic inputs, and maintaining the specificity of synaptic connections, reducing signal interference. These finding challenges previous studies that suggest dendritic arborization occurs randomly [38,39]. Many earlier studies focused on single dendritic trees, failing to account for the influence of neighboring branches. Consequently, past conclusions regarding random arborization may have overlooked the role of branch-to-branch interactions in shaping dendritic structure.

**Fig 10:**
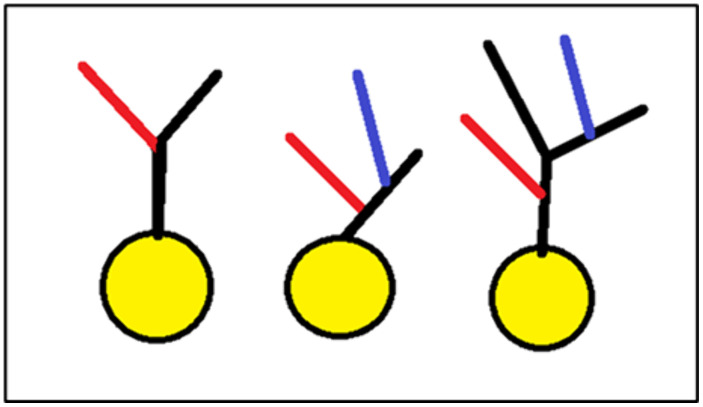
Branch-to-branch parallel growth-based dendritic morphogenesis. A model describing the emergence of specific harbor configurations of neighboring dendrites due to parallel growth among their branches. Parallel branch growth is indicated by color. Black – non-parallel growth. Blue and red – parallel.

This model is illustrated in Fig 10 and highlights the importance of considering interactions between multiple dendritic trees rather than analyzing individual trees in isolation. By incorporating these broader network effects, we gain a more accurate understanding of dendritic patterning and neuronal circuit formation.

While previous studies of dendritic morphogenesis have focused on isolated neurons in simplified or sparsely cultured conditions, our results suggest that the spatial configuration of neighboring dendritic branches, particularly their parallel alignment, may represent a biologically meaningful pattern that arises through inter-neuronal coordination. This observation challenges conventional morphometric analyses that treat dendrites as independent structures and emphasizes the need for tools that capture network level organization.

Our findings align with recent insights from morphological modeling and computational neuroscience, which suggest that dendritic arborization is not solely governed by cell intrinsic cues, but also shaped by extrinsic spatial constraints and intercellular interactions [40,41]. The presence of coordinated, parallel growth patterns in our 2D culture system suggests that dendritic structures cannot be fully understood when studied in isolation. Instead, quantitative methods must incorporate the broader context of the neuronal population to interpret arborization strategies accurately.

By framing dendritic growth as a collective, rather than solitary, process, this study contributes to a growing body of literature advocating for population-level and network-aware morphometric analyses. Future work should investigate whether similar patterns are observed in vivo and identify the molecular and physical mechanisms that promote such spatial coordination. These findings open new avenues for exploring how developmental constraints and environmental cues shape dendritic organization in both physiological and pathological contexts.

## Conclusion

The phenomenon of parallel dendritic growth is a highly regulated process influenced by both intrinsic and extrinsic factors. The ability of both sister and non-sister branches to align in parallel, the spatial constraints on this growth, and the role of proximity highlight the intricate coordination required for proper dendritic development. Understanding these mechanisms is crucial for unraveling the principles of neural circuit formation and for identifying potential disruptions in neurodevelopmental disorders.

Our study shifts the focus from isolated dendritic analysis to the collective behavior of branches within neuronal networks. By utilizing SOA 2.0, an innovative tool designed for quantifying dendritic morphology, we demonstrated that parallel growth is not a random occurrence but a prevalent organizational feature essential for neuronal architecture.

These findings have implications that extend beyond basic neuroscience. Disruptions in dendritic growth have been linked to neurodevelopmental and neurodegenerative disorders, suggesting that understanding the mechanisms regulating parallel growth could reveal therapeutic targets for impaired neural circuits. Future research should focus on identifying the molecular cues and signaling pathways that drive this growth and exploring its role in synaptic integration, memory formation, and learning. By further elucidating these principles, we can advance our understanding of neuronal network development and function, with potential applications in both basic research and clinical neuroscience.

## Supporting information

S1 Table Data display collection of all the analysis images. For each file image, the table provides data on the number of detached branches, the number of parallel groups and lines observed in both the measured and simulated datasets, as well as the calculated parallelism percentages. Additionally, it presents the Group-Weighted Parallelism ratio (E/S).
(TIF)

S2 Table Comprehensive summary statistics. The described data gives us the count, mean, standard deviation (std), minimum, Q1 (25%), median (50%), Q3 (75%), IQR (Q3 - Q1) and maximum values.
(TIF)

S3 Table Analysis of astrocyte images. This table summarizes the results of the parallelism analysis performed on seven astrocyte images using the SOA.2.0 software. For each image, the number of detached branches, number of parallel groups and lines (both measured and simulated), and the percentage of parallelism were calculated. The measured parallelism percentages are compared to the simulated values, and ratios are presented to quantify the deviation from random expectations. Notably, all measured values show lower parallelism than their simulated counterparts. The final column presents the long-term weights ratio (E/S), further highlighting the reduced organization in astrocyte branching compared to dendritic cultures. To statistically assess whether this observed difference was significant, we performed a Mann–Whitney U test comparing the distribution of measured parallelism in astrocytes to the corresponding simulated values. The test yielded a p-value of 0.007. These findings suggest that the pronounced parallel alignment detected in dendritic cultures is unlikely to be an artifact of the analysis pipeline and may instead reflect a biologically meaningful organizational pattern specific to neuronal structures.
(JPG)

S4 Table Analysis of NeuroMorpho.Org images. This table summarizes the results of the parallelism analysis performed on neuronal images obtained from the public NeuroMorpho.Org repository using the SOA.2.0 software. For each image, the number of detached branches, number of parallel groups and lines (both measured and simulated), and the percentage of parallelism were calculated. The measured parallelism percentages were compared with simulated values, and ratios were computed to assess deviation from random expectations. In both images, the measured parallelism was slightly lower than the simulated baseline, with an average measured-to-simulated ratio of 0.88 and an E/S weight ratio of 0.95. These results indicate a predominantly random orientation of dendritic branches in isolated neuronal reconstructions, reinforcing that the strong parallel alignment observed in dendritic cultures is not an artifact of the analysis pipeline.
(TIF)
