# Supplementary figures and images for: A method for quantifying parallel growth between neuronal dendritic branches in vitro
Source: PLoS One. 2025 Oct 31;20(10):e0335919. doi: 10.1371/journal.pone.0335919 (PMC12578326; doi:10.1371/journal.pone.0335919)

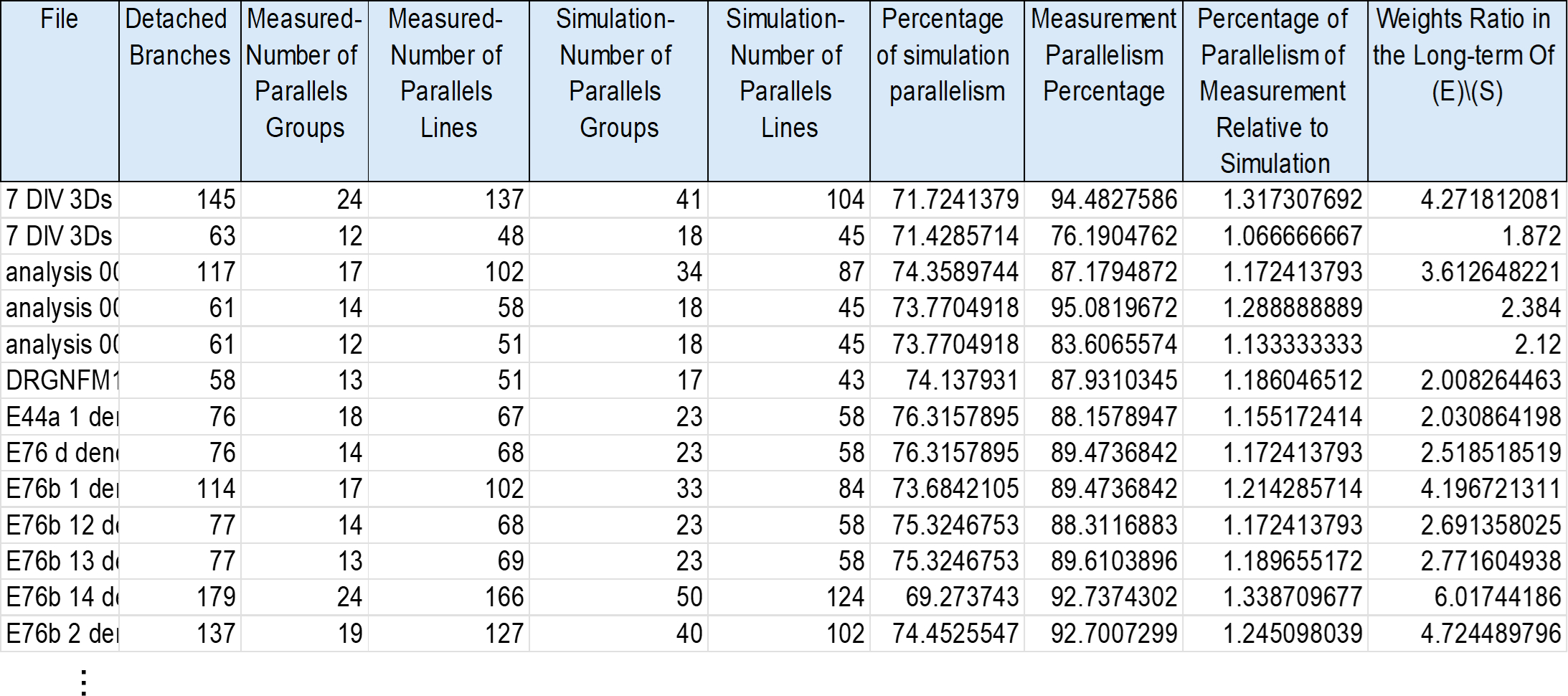

Supplement: S1 Table — For each file image, the table provides data on the number of detached branches, the number of parallel groups and lines observed in both the measured and simulated datasets, as well as the calculated parallelism percentages. Additionally, it presents the Group-Weighted Parallelism ratio (E/S). (TIF) [file pone.0335919.s001.tif]

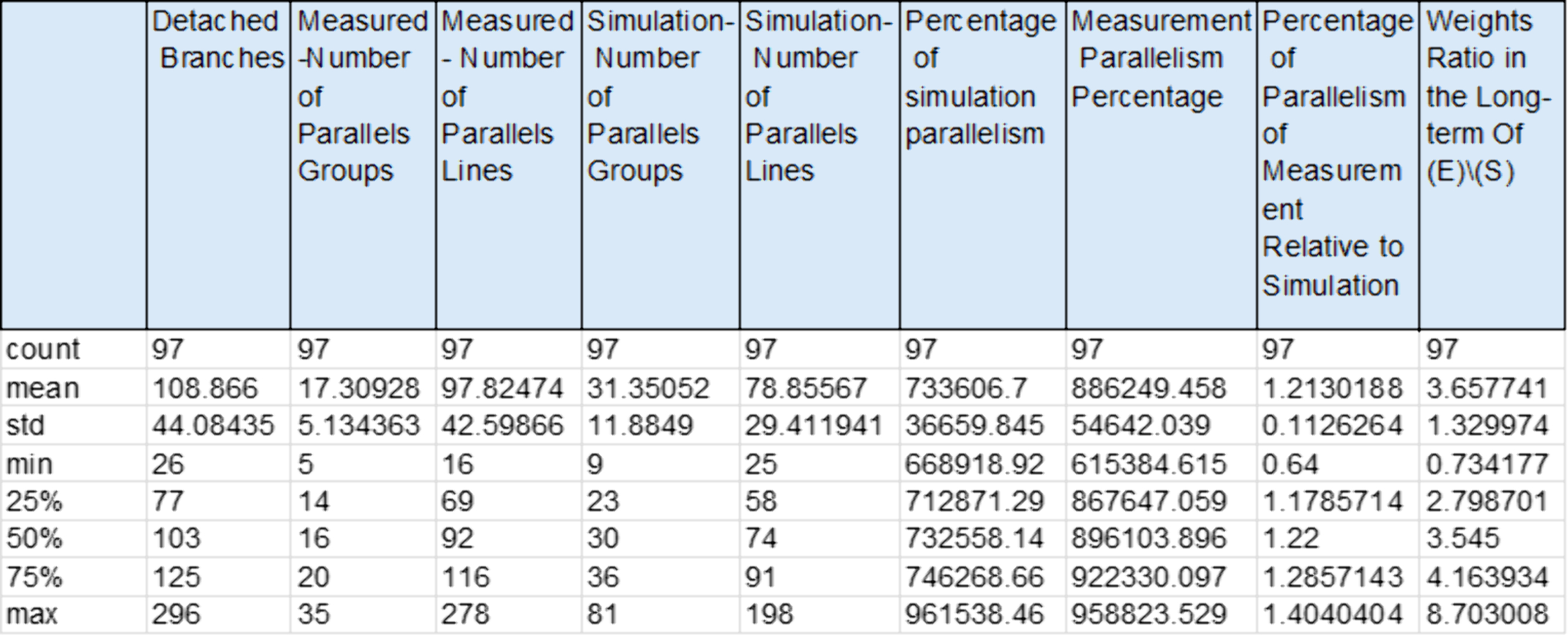

Supplement: S2 Table — The described data gives us the count, mean, standard deviation (std), minimum, Q1 (25%), median (50%), Q3 (75%), IQR (Q3 - Q1) and maximum values. (TIF) [file pone.0335919.s002.tif]

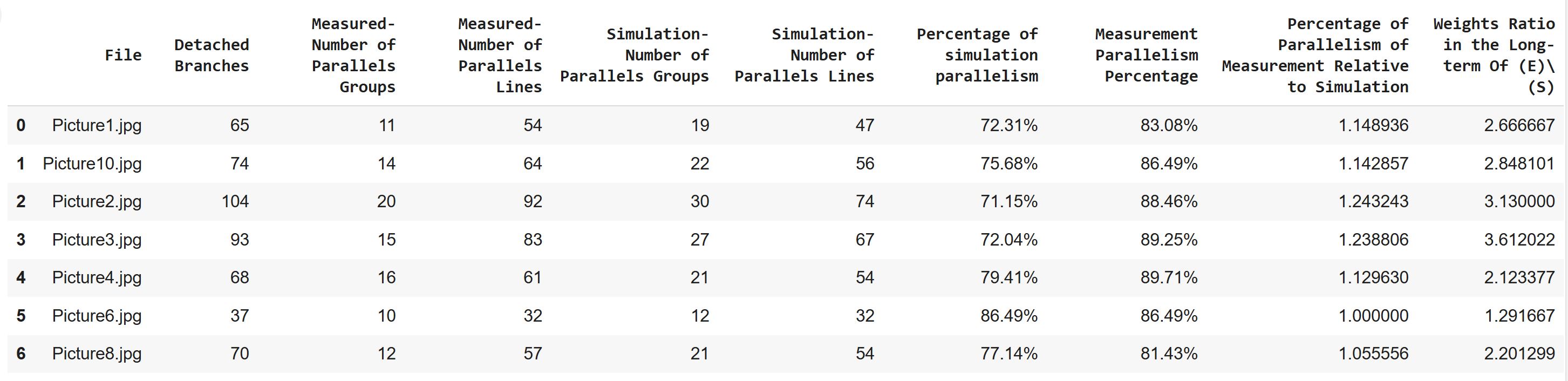

Supplement: S3 Table — This table summarizes the results of the parallelism analysis performed on seven astrocyte images using the SOA.2.0 software. For each image, the number of detached branches, number of parallel groups and lines (both measured and simulated), and the percentage of parallelism were calculated. The measured parallelism percentages are compared to the simulated values, and ratios are presented to quantify the deviation from random expectations. Notably, all measured values show lower parallelism than their simulated counterparts. The final column presents the long-term weights ratio (E/S), further highlighting the reduced organization in astrocyte branching compared to dendritic cultures. To statistically assess whether this observed difference was significant, we performed a Mann–Whitney U test comparing the distribution of measured parallelism in astrocytes to the corresponding simulated values. The test yielded a p-value of 0.007. These findings suggest that the pronounced parallel alignment detected in dendritic cultures is unlikely to be an artifact of the analysis pipeline and may instead reflect a biologically meaningful organizational pattern specific to neuronal structures. (JPG) [file pone.0335919.s003.JPG]

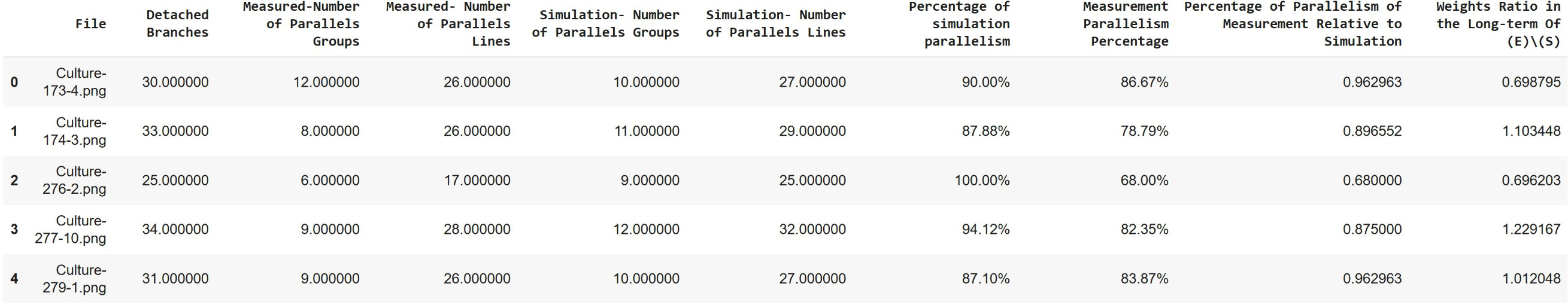

Supplement: S4 Table — This table summarizes the results of the parallelism analysis performed on neuronal images obtained from the public NeuroMorpho.Org repository using the SOA.2.0 software. For each image, the number of detached branches, number of parallel groups and lines (both measured and simulated), and the percentage of parallelism were calculated. The measured parallelism percentages were compared with simulated values, and ratios were computed to assess deviation from random expectations. In both images, the measured parallelism was slightly lower than the simulated baseline, with an average measured-to-simulated ratio of 0.88 and an E/S weight ratio of 0.95. These results indicate a predominantly random orientation of dendritic branches in isolated neuronal reconstructions, reinforcing that the strong parallel alignment observed in dendritic cultures is not an artifact of the analysis pipeline. (TIF) [file pone.0335919.s004.tif]
